# Roles for Coincidence Detection in Coding Amplitude-Modulated Sounds

**DOI:** 10.1371/journal.pcbi.1004997

**Published:** 2016-06-20

**Authors:** Go Ashida, Jutta Kretzberg, Daniel J. Tollin

**Affiliations:** 1 Cluster of Excellence "Hearing4all", Department for Neuroscience, Faculty 6, University of Oldenburg, Oldenburg, Germany; 2 Department of Physiology and Biophysics, University of Colorado School of Medicine, Aurora, Colorado, United States of America; Carnegie Mellon University, UNITED STATES

## Abstract

Many sensory neurons encode temporal information by detecting coincident arrivals of synaptic inputs. In the mammalian auditory brainstem, binaural neurons of the medial superior olive (MSO) are known to act as coincidence detectors, whereas in the lateral superior olive (LSO) roles of coincidence detection have remained unclear. LSO neurons receive excitatory and inhibitory inputs driven by ipsilateral and contralateral acoustic stimuli, respectively, and vary their output spike rates according to interaural level differences. In addition, LSO neurons are also sensitive to binaural phase differences of low-frequency tones and envelopes of amplitude-modulated (AM) sounds. Previous physiological recordings *in vivo* found considerable variations in monaural AM-tuning across neurons. To investigate the underlying mechanisms of the observed temporal tuning properties of LSO and their sources of variability, we used a simple coincidence counting model and examined how specific parameters of coincidence detection affect monaural and binaural AM coding. Spike rates and phase-locking of evoked excitatory and spontaneous inhibitory inputs had only minor effects on LSO output to monaural AM inputs. In contrast, the coincidence threshold of the model neuron affected both the overall spike rates and the half-peak positions of the AM-tuning curve, whereas the width of the coincidence window merely influenced the output spike rates. The duration of the refractory period affected only the low-frequency portion of the monaural AM-tuning curve. Unlike monaural AM coding, temporal factors, such as the coincidence window and the effective duration of inhibition, played a major role in determining the trough positions of simulated binaural phase-response curves. In addition, empirically-observed level-dependence of binaural phase-coding was reproduced in the framework of our minimalistic coincidence counting model. These modeling results suggest that coincidence detection of excitatory and inhibitory synaptic inputs is essential for LSO neurons to encode both monaural and binaural AM sounds.

## Author Summary

Detecting coincident arrivals of synaptic inputs is a shared fundamental property of many sensory neurons. Such 'coincidence detection' usually refers to the detection of synchronized excitatory inputs only. Experimental evidence, however, indicated that some auditory neurons are also sensitive to the relative timing of excitatory and inhibitory synaptic inputs. This type of sensitivity is suggested to be important for coding temporal information of amplitude-modulated sounds, such as speech and other naturalistic sounds. In this study, we used a minimal model of coincidence detection to identify the key elements for temporal information processing. Our series of simulations demonstrated that (1) the threshold and time window for coincidence detection were the major factors for determining the response properties to excitatory inputs, and that (2) timed interactions between excitatory and inhibitory synaptic inputs are responsible for determining the temporal tuning properties of the neuron. These results suggest that coincidence detection is an essential function of neurons that detect the 'anti-coincidence' of excitatory and inhibitory inputs to encode temporal information of sounds.

## Introduction

Sound source localization, or the ability to determine the direction of a sound source, is a fundamental function of the auditory system. The interaural level difference (ILD), which is the difference of the sound levels between the two ears, is one of the primary cues of azimuthal sound localization (e.g., [1]). ILDs generally increase with sound frequency and source azimuth [2,3]. In the mammalian auditory brainstem, ILDs are first encoded by neurons of the lateral superior olive (LSO) [4,5] (see [6] for a review). Principal neurons in the LSO receive excitatory inputs from the bushy cells in the ipsilateral ventral cochlear nucleus (VCN) [7–11] and inhibitory inputs from the medial nucleus of the trapezoid body (MNTB) driven by contralateral sound stimuli [8,9,12,13]. Consequently, the binaural excitatory-inhibitory interaction at the LSO is the source of the ILD-dependent spike-rate coding, which has been a subject of numerous modeling studies (e.g., [14–19]). Furthermore, because of the interaction between phase-locked excitatory [20,21] and inhibitory inputs [22,23], spike rates of LSO neurons also vary with the interaural time difference (ITD) of low-frequency tones [24–26] or of the envelopes of amplitude-modulated (AM) sounds [27–29]. Importance of temporally precise excitatory-inhibitory interactions was also suggested by measured LSO responses to transient stimuli such as clicks [27,30].

Amplitude modulation is a general feature of natural stimuli including communication sounds, and hence processing of AM signals is a fundamental property of the auditory system [31]. For example, the critical importance for AM in speech perception has been demonstrated in a variety of studies where degradation of the modulation spectra results in loss of intelligibility (e.g., [32]). Moreover, physiological studies have shown that neurons at different levels of the ascending auditory neuraxis acquire sensitivities to AM stimuli that are different from those seen at more peripheral levels of the auditory system [33], and many of these neurons also exhibit sensitivity to the binaural cues to sound location [34]. It has been suggested that the sensitivities of more central auditory centers to specific ranges of AM reflect the necessity to encode ecologically relevant aspects of the acoustic environment (e.g., [35]).

**Fig 1:**
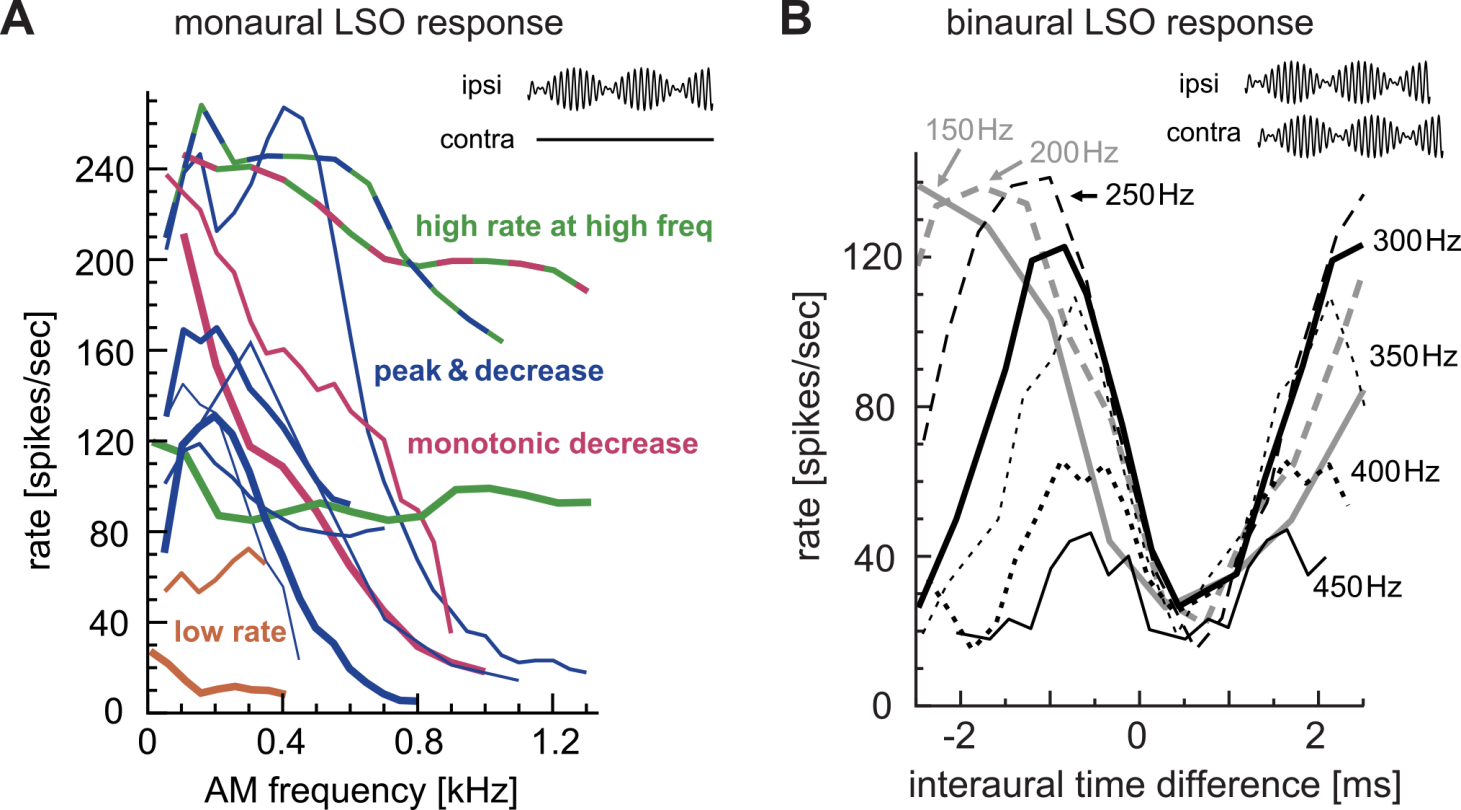
Recorded responses of cat LSO neurons to AM sounds. **A:** Monaural AM responses with varied modulation frequencies. Different lines are used for different LSO units. Several response types of AM-tuning were found and shown in different colors. Some units exhibited characteristics of multiple response types. **B:** Binaural AM responses with varied ITDs. Different line types correspond to different modulation frequencies. Adapted and redrawn from Figs 13B and 16B of Joris and Yin [36] with permission.

LSO responses to monaural AM tones at different modulation frequencies were systematically studied by Joris and Yin [36]. Typically, spike rates showed a mild peak at modulation frequencies (*f*_*m*_) of 100–500 Hz, and gradually decreased down to 50 spikes/sec at around *f*_*m*_ = 600–1000 Hz (Fig 1A, blue lines). However, the variability of responses from neuron to neuron was unexpectedly large. Some LSO neurons showed monotonic decreases in spike rate along the modulation-frequency axis (Fig 1A, red lines) whereas spike rates of other units remained > 100 spikes/sec over the *f*_*m*_ ranges tested (Fig 1A, green lines). A few LSO neurons showed low spike rates for monaural AM sounds (Fig 1A, orange lines). Joris and Yin [36] suggested that the low-pass nature of LSO rate tuning was likely not inherited from their inputs from spherical bushy cell in the VCN, because the firing rates of bushy cells were more stable (i.e., all-pass) with increasing *f*_*m*_ than LSO neurons. The source of the variability in AM rate coding should thus originate at the synaptic and membrane levels of the LSO neuron, making a contrast to frequency tuning in the inferior colliculus that inherits and combines a variation of spectral tuning patterns of ascending projections [37,38]. Wang and Colburn [39] studied LSO responses to AM sounds, using detailed conductance-based models. Their series of simulations suggested that the membrane afterhyperpolarization, which was shown to be important for the characteristic ‘chopping’ responses of LSO to pure tone stimuli [18], was unlikely to be the primary mechanism for rate decreases in AM coding with increasing *f*_*m*_, whereas the addition of a large amount of low-voltage-activated potassium (KLVA) conductance led to rate-*f*_*m*_ functions that were more consistent with empirical results. Nevertheless, no combinations of parameters were able to comprehensively explain the diversity of LSO responses to monaural AM stimuli.

Previous physiological recordings *in vivo* revealed a number of characteristic response properties of LSO neurons to binaural AM sounds [27–29,36]. The output spike rate of an LSO neuron typically shows periodic changes with ITDs of the envelope of AM sounds, and its period equals the reciprocal of the modulation frequency *f*_*m*_ (Fig 1B). Furthermore, ITD-tuning curves measured at different *f*_*m*_ align at their toughs (Fig 1B), which is considered as a signature of excitatory-inhibitory interactions [27,29]. Since prior modeling approaches focused predominantly on intensity (ILD) coding (e.g., [14–18]), how LSO neurons encode binaural temporal information remained largely uncovered.

In the classical view of binaural coding in the brainstem, LSO neurons encode ILDs by simply subtracting inhibitory inputs from excitatory inputs (reviewed in [1,6]), whereas in the medial superior olive (MSO: another major nucleus in the mammalian auditory brainstem) coincidence detection of binaural excitatory inputs is essential for encoding ITDs [1,40,41]. However, monaural and binaural AM-tunings of LSO suggest that LSO neurons should also perform temporally precise processing of bilateral synaptic inputs. Since LSO neurons receive ipsilaterally driven excitatory inputs and contralaterally driven inhibitory inputs, binaural processing in the LSO is expected to be 'anticoincidence detection', in which the response rate of the neuron becomes maximal when its excitatory and inhibitory synaptic inputs arrive temporally out-of-phase.

In order to reveal underlying mechanisms of monaural and binaural AM coding in the LSO, we use a simple model of coincidence detection, which is based on the counting of coincident inputs [42,43]. Because of the smaller number of free parameters than previous conductance-based LSO models (e.g., [39,44,45]), our model allows us to examine the contribution of each biophysical factor in a much more simplified situation. After identifying the factors responsible for the observed unit-to-unit variability in monaural AM coding, we examine how these factors affect the temporal interaction of excitatory and inhibitory inputs and resulting response properties of the LSO neuron to binaural AM sounds.

## Materials and Methods

### Excitatory Inputs to LSO

Bushy cells in the VCN generally show phase-locked spiking activities to the envelope of AM sound stimuli. Excitatory phase-locked synaptic inputs to the LSO were modeled as an inhomogeneous Poisson process [46,47]. The output spike rate of each bushy cell was described as a periodic function:

$$\lambda (t)=2\pi \bar{\lambda }p(2\pi f_{m}t),$$

where *t* is time, $\bar{\lambda }$ is the average intensity, *p* is a 2π-periodic function, and *f*_*m*_ is the stimulus modulation frequency. We used a von-Mises distribution function [48] for the periodic function *p*(*x*). Assuming that I_n_ is the Modified Bessel function of order n, the periodic function we used is written as:

$$p_{k}(x)=\exp(k\cos(x))/(2\pi I_{0}(k)),$$

with *k* being the concentration parameter that defines the steepness of the distribution. The degree of phase-locking, measured as vector strength (VS) [49], is related to the concentration parameter as:

$$VS=I_{1}(k)/I_{0}(k).$$

For more detail about theoretical formulations, see [47].

**Fig 2:**
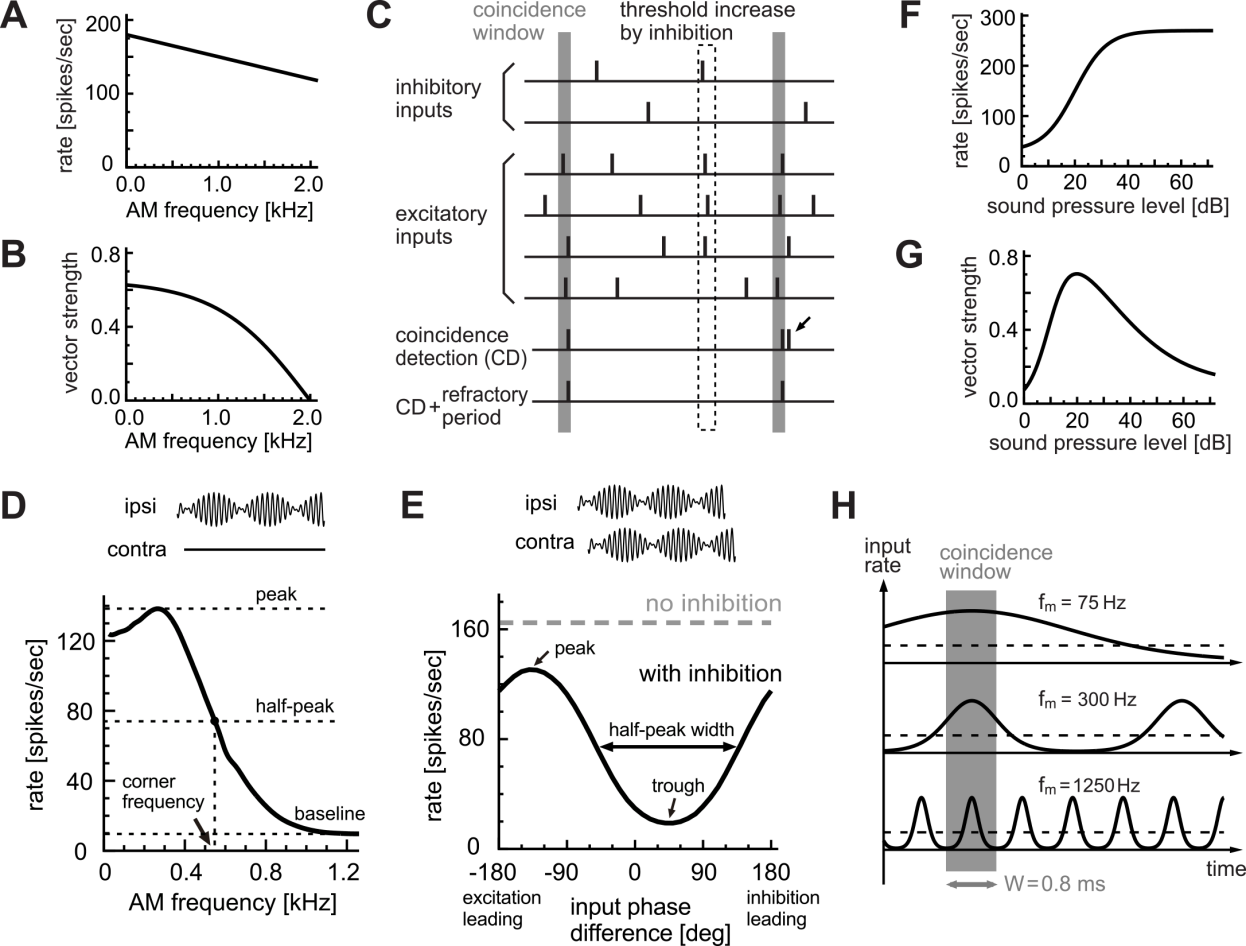
LSO coincidence counting model. **A:** Modeled AM-frequency dependence of the excitatory input rates. **B:** Modeled AM-frequency dependence of phase-locking of excitatory inputs. **C:** Modeled operation of coincidence detection. Each vertical bar corresponds to a spike. An input coincidence is counted when the number of inputs in the coincidence window W (vertical gray rectangle) reaches or exceeds the threshold θ. In this example, threshold θ is 3. The small arrow indicates an output spike rejected by the refractory period T. Effects of inhibitory inputs were modeled as threshold increase δ in the inhibition window Δ (dotted rectangle). **D:** Monaural AM-tuning curve with the default parameter set (θ = 8 inputs, W = 0.8 ms, T = 1.6 ms). Peak rate = 138.3 spikes/sec. Peak frequency = 265 Hz. Baseline = 9.7 spikes/sec. Half-peak frequency = 549 Hz. **E:** Binaural AM-phase coding with the default parameter set (*f*_*m*_ = 300 Hz, δ = 2 inputs and Δ = 1.6 ms). Peak rate = 130.7 spikes/sec. Peak phase = −137 deg. Trough rate = 18.7 spikes/sec. Trough phase = +46 deg. Half-peak width = 191 deg. **F:** Modeled level-dependence of input spike rates. G: Modeled level-dependence of phase-locking. Both excitatory and inhibitory inputs were assumed to share the rate-level and VS-level functions. **H:** Effects of modulation frequency *f*_*m*_ on coincidence detection. Depending on frequency, the length of one modulation cycle could be larger (at low *f*_*m*_) or smaller (at high *f*_*m*_) than width W of the coincidence window. Thick curves show the spike rates of the phase-locked inputs. Broken lines show the time-averaged (non-phase-locked) spike rates.

The average spike rate and the VS of a bushy cell to AM stimuli generally decrease with the modulation frequency (gerbil: [20]; cat: [21,36]). Based on previous physiological measurements, we modeled them as:

$$\bar{\lambda }(f_{m})=\lambda_{0}-0.03f_{m}\;\left(\text{spikes}/\text{sec}\right),$$

and

$$\text{VS}(f_{m})=0.65\;(1-\exp((f_{m}-2000)/500))/(1+\exp((f_{m}-2000)/500))\;(f_{m}<2000),$$

with *f*_*m*_ being the modulation frequency in Hz. Since there were considerable variations in bushy cell responses between animal species and between experimental conditions, we used a simple linear function for the rate (Fig 2A) and a monotonic decreasing function for the VS (Fig 2B), both of which roughly mimic the recorded response properties [20,21,36]. The default value of λ_0_ (input intensity at zero modulation frequency) was 180 (spikes/sec). The non-linear decreasing function for simulating VS reaches zero at *f*_*m*_ = 2000 Hz. In our simulations, we used the above-defined rate and VS functions unless otherwise stated. As in a previous study [39], we assumed that an LSO neuron receives 20 excitatory inputs that are locked to the same stimulus phase (see also caption of Table 1).

**Table 1:** Model Parameters. (#1) Sanes [51] reported that the number of excitatory subthreshold inputs was 9.6 ± 2.8 in gerbils, which serves as the lower bound of the total number of excitatory inputs. The MSO neuron, which has a similar anatomical structure to the LSO neuron, receives a few times more excitatory inputs than inhibitory inputs [53]. Assuming a similar ratio, 20 excitatory inputs (derived from 8 inhibitory inputs) would be reasonable. (#2) Measured membrane time constants of gerbil LSO cells were 1.1 ± 0.4 ms and minimum durations of excitatory synaptic inputs were 1.5 ± 0.8 ms [51]. We assumed that these values roughly limit the maximum width of the coincidence window. (#3) Based on the measured minimum durations of inhibitory synaptic inputs of 3.2 ± 1.7 ms in gerbils [51], we assumed the inhibition window to be twice as long as the coincidence window. (#4) As far as we know, there is no direct measurement available. See Discussion for more information on relevant experimental values.

Parameter	Default value	Simulated range	Relevant experimental values
Number of excitatory inputs M_ex_	20	(fixed)	(#1)
Number of inhibitory inputs M_inh_	8	(fixed)	8.2 ± 3.5 (gerbils) [51]
Coincidence threshold θ	8	5–11	9.6 ± 2.8 (gerbils) [51]
Coincidence window W	0.8 ms	0.4–1.3 ms	(#2)
Refractory period T	1.6 ms	0.8–2.4 ms	1.1–2.8 ms (cats) [52]
Inhibition window Δ	1.6 ms	0.8–2.4 ms	0.8–2.0 ms (rats) [30] (#3)
Threshold increase δ	2	0–8	(#4)

### Inhibitory Inputs to LSO

Even without sound stimuli, LSO neurons receive spontaneous inhibition from MNTB neurons [23,50]. Based on these previous measurements, we modeled the spontaneous inhibitory inputs as a (homogeneous) Poisson process with a fixed intensity of *λ*_inh_ = 30 (spikes/sec). We assumed that an LSO neuron receives 8 inhibitory inputs [51]. For testing binaural phase-coding of the model LSO, sound-evoked inhibitory inputs from MNTB neurons were similarly modeled as the excitatory inputs from bushy cells described above, simply using the same intensity and phase-locking parameters.

### Coincidence Counting Model of LSO

Coincidence detection in the LSO was modeled as simple coincidence counting (Fig 2C), which was introduced in a previous study of binaural coincidence detection in the MSO [43]. A coincidence window of size W (vertical gray band in Fig 2C) slides along the time axis. When the number of input spikes in the coincidence window reached or exceeded the pre-set threshold θ, an output spike was generated (θ = 3 in Fig 2C). If more than one output spike occurred within the pre-set refractory period T, then only the first spike was counted and the other spikes were discarded (small arrow in Fig 2C). Effects of inhibition were modeled as a temporary increase in the threshold. Namely, when the model neuron received an inhibitory input at time *t*, the coincidence threshold between time *t* and *t+*Δ was elevated by δ. We refer to this effective length Δ of the inhibition as 'inhibition window' and to the elevation δ of the threshold as 'threshold increase'. Standard parameter values are summarized in Table 1 and explained in the next section.

### Model Coincidence Parameters

Our coincidence counting model has three fundamental parameters: the coincidence threshold θ, the width of the coincidence window W, and the length of the refractory period T. The default values we used were: θ = 8 inputs, W = 0.8 ms, and T = 1.6 ms, which were taken from empirically measured parameter ranges (Table 1; see also Discussion) to reproduce modulation-frequency tuning observed in LSO neurons (Fig 2D; see Fig 1A and [36] for experimental results). We varied these parameters independently and examined their effects on the monaural AM coding.

To test combined effects of the coincidence threshold and window, we varied these parameters together while fixing the ratio W/θ, so that the average number of required inputs within the window remains unchanged. We also covaried these parameters so that the maximum response rate was constant (see corresponding sections in Results for detail).

For simulating binaural AM coding, we assumed that Δ = 1.6 ms and δ = 2, which reproduces empirical phase-coding results of LSO (Fig 2E; see Fig 1B and [27] for experimental results). The relative widths of the coincidence window W and the inhibition window Δ also reflect previous slice recording results, in which the duration of an inhibitory postsynaptic current was roughly twice as long as that of an excitatory synaptic current [51]. In our numerical simulations, we used a time step of 2 μs; results were not affected by the choice of the time step if it was equal to or smaller than this value.

### Monaural Tuning: Peak, Half-Peak and Baseline

Simulated output spike rates of the LSO model in response to monaural AM inputs depended on the modulation frequency. An 'AM-tuning curve' generally showed a peak at a certain frequency and decreases with increasing modulation frequency (Fig 2D). Following a notation of prior studies (e.g., [31,54]), we also refer to the rate-*f*_*m*_ relation shown by an AM-tuning curve as the 'rate modulation transfer function', or the 'rate-MTF'.

To characterize the modulation-frequency dependence of the LSO output, we calculated the peak rate, peak AM frequency, and the baseline rate of the rate-MTF. The baseline rate is defined as the lowest spike rate for 25–1200 Hz. We also calculated the position of the half-peak rate, where the simulated spike rate reaches 50% of the peak rate measured from the baseline (Fig 2D). We refer to the frequency position of the half-peak rate as the 'corner frequency'. For each parameter set, we simulated the input spike sequences over 100 seconds and calculated the average output spike rate of the model LSO neuron. To estimate peak frequencies, we applied five-point smoothing and spline-interpolation to the rate-MTF curve.

### Phase-Locking

Phase-locking of the LSO responses was quantified by the modulation gain, as was done in a previous experimental study [36]. The modulation gain is defined by 20log_10_(2*R*), where *R* is the VS of the LSO output spike sequence at the modulation frequency *f*_*m*_. Following a notation of prior studies (e.g., [54]), we also refer to the gain-*f*_*m*_ relation as the 'synchrony modulation transfer function', or the 'synch-MTF'.

### Binaural Tuning: Peak, Trough and Half-Peak

Simulated output spike rates of the LSO model in response to binaural AM inputs generally depended on the phase differences of excitatory and inhibitory inputs. A 'phase-tuning curve' generally showed a peak and a trough at certain phase differences (Fig 2E). As in previous experimental studies [26,27], we defined a positive phase difference as inhibitory inputs preceding excitatory inputs. To characterize the phase dependence of the model LSO output, we varied the phase differences of simulated excitatory and inhibitory inputs and calculated the average output spike rate of the model LSO neuron over 100 seconds at each phase difference. To examine how the model parameters (i.e., coincidence parameters θ, W, T and inhibition parameters Δ, δ) affect the binaural phase coding, we calculated the peak and trough rates, trough phase, and the half-peak width of the phase-tuning curve (Fig 2E). We used the above-described default parameters (summarized in Table 1) throughout our simulations unless otherwise stated. For testing the effects of coincidence parameters, we fixed the AM frequency at *f*_*m*_ = 300 Hz, which roughly corresponded to the peak of the monaural AM response (Fig 2D), and varied the relative input timings of excitatory and inhibitory inputs. For testing frequency dependence of binaural tuning, we varied the AM frequency at 150, 300 450 and 600 Hz.

### Effects of Sound Levels

LSO neurons are sensitive to ILDs [4–6], and hence the phase-tuning curve of an LSO neuron is also affected by ILD [27]. To examine the ILD-dependence of binaural AM coding, we introduced level dependence to the intensity of excitatory and inhibitory inputs. Although there were noticeable variations across species, spike rates of input fibers generally increase with sound level (cat VCN: [36,55]; monkey VCN: [56]; gerbil VCN: [57]; cat MNTB: [22,50]; rodent MNTB: [23]). Based on these measurements, we used a sigmoid function to roughly approximate the level-dependence of these input fibers:

$$\bar{\lambda }(l)=30+240/(1+\exp(-(l-20)/6.0))\;\left(\text{spikes}/\text{sec}\right),$$

where *l* is the sound pressure level in dB (see Fig 2F for the curve shape). Since VCN bushy cells and MNTB neurons show similar level dependence, we assumed that both excitatory and inhibitory inputs share this level-dependence function. Non-monotonic, non-linear level-dependence of the degree of synchrony (VS) [21,36] was modeled as a combination of two sigmoid functions:

VS(l)=(1.0/(1+exp(−(l−10)/4.0)))×(0.1+1.0/(1+exp(+(l−30)/15.0))),

with *l* being the level in dB. The first and second terms correspond to the increasing (at low levels) and decreasing (at high levels) parts of the curve, respectively (Fig 2G).

The modeled ILDs were varied in our simulation. Positive ILD indicates that the contralateral sound that drives inhibitory inputs is stronger than the ipsilateral sound that drives excitatory inputs. Note that the level-dependent input rates were only used for the figure where effects of level differences are shown. For other simulation results, we assumed that the sound level was fixed and used the *f*_*m*_-dependent input rates described earlier.

### Pure Integrator Model

To clarify the importance of coincidence detection in LSO, we also created a 'pure integrator' model that lacks temporal windows but simply sums up the number of synaptic inputs irrespectively of their timings. Let N_e_(*t*) and N_i_(*t*) be the numbers of excitatory and inhibitory inputs, respectively, that the model neuron received between the end of the last refractory period and time *t*. When the summed excitatory spike counts subtracted by the weighted number of inhibitory inputs reached or exceeded the pre-set threshold θ, one output spike was generated and the model was then in a refractory period T. Namely, a spike was generated at time *t*, when N_e_(*t*) - δN_i_(*t*) ≥ θ. The weight δ of the inhibitory input corresponds to the threshold increase by inhibition. Thus the pure integrator model has three parameters: threshold θ, refractory period T and threshold increase δ. We used the same default values for this model (θ = 8 inputs, T = 1.6 ms, δ = 2 inputs) as for the coincidence counting model (Table 1).

## Results

### Monaural AM Coding in LSO

#### Basic mechanisms

With the default parameter set (see Materials and Methods), the simulated monaural rate-MTF curve (Fig 2D) showed a small peak around 200–300 Hz, similar to empirical *in vivo* results (Fig 1A, blue lines). The modeled spike rate was generally higher for low modulation frequencies than for high. Fig 2H depicts the basic mechanism of this frequency dependence. For modulation frequencies that are slightly smaller than a half of the reciprocal of the coincidence window (Fig 2H, middle), the average number of inputs within the coincidence window varies considerably with time, resulting in a higher number of coincidences counted. For higher modulation frequencies (Fig 2H, bottom), the number of excitatory inputs stays almost constant (remains subthreshold) irrespective of the temporal location of the coincidence window, leading to a low number of output spikes. In other words, the temporal variation of the input counts within the coincidence window is not large enough to evoke a spike. For very low frequencies (Fig 2H, top), more than one coincidence may be counted within a modulation cycle, but some of them are rejected by the refractory period, resulting in slightly lower output rate. The peak frequency of the rate-MTF curve depends on the model parameters, which will be discussed in the following subsections.

#### Effects of excitatory inputs

**Fig 3:**
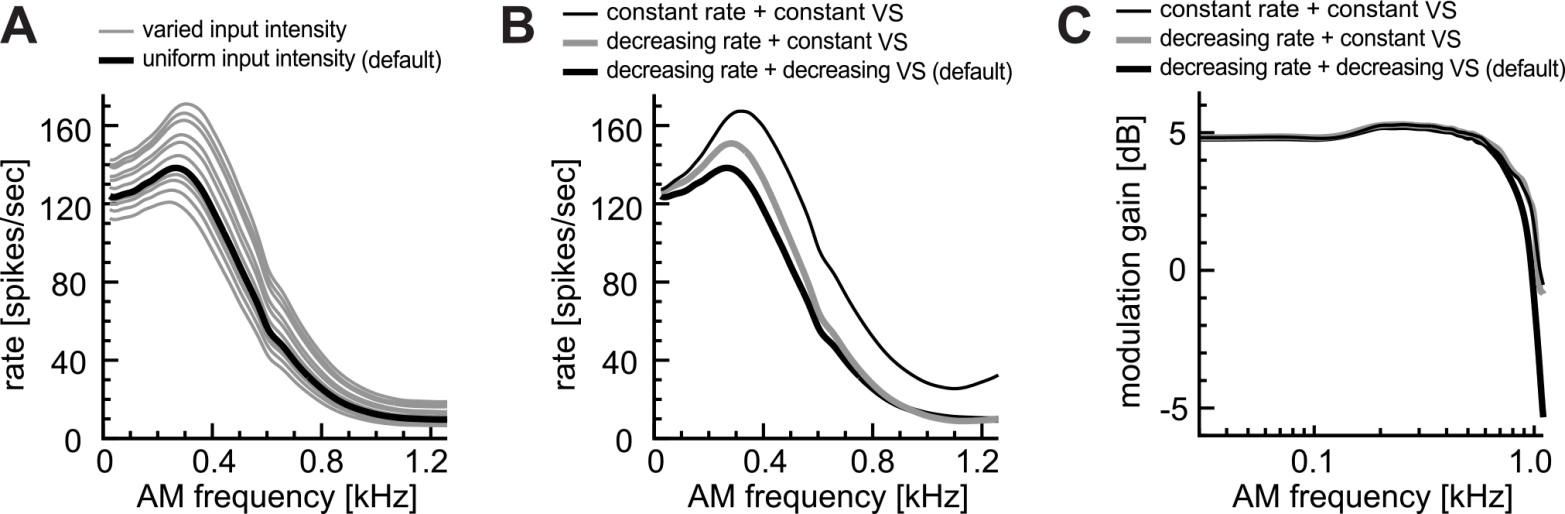
Effects of excitatory inputs. **A:** Effects of non-uniform input intensities. **(Black curve: uniform intensity)** The default input intensity of λ_0_ = 180 spikes/sec (at zero modulation frequency) was used for all input fibers. **(Gray curves: varied intensity)** The input intensity λ_0_ of each fiber was randomly chosen from a uniform distribution between 80 and 280 spikes/sec. Results for ten simulation trials are shown. **B:** Effects of input parameters on spike rates. In the 'constant rate' condition, the average input rate $\bar{\lambda }(f_{m})$ was fixed at 180 spikes/sec. In the 'constant VS' condition, VS was fixed to 0.65. Otherwise, these parameters were varied with the modulation frequency (see Materials and Methods). **C:** Effects of input parameters on the modulation gain. Line types in C correspond to those in B.

First, we tested how variations in input parameters may alter the rate-MTF (Fig 3). Variations of the intensity λ_0_ of excitatory input fibers slightly modified the overall output rate of the model LSO, but the shape of the rate-MTF curve was not greatly affected (Fig 3A). Therefore, in our following simulations, we assumed that all input fibers have the same rate λ_0_ (see Materials and Methods for equations). The modulation-frequency dependence of input spike rate and phase-locking also did not generally affect the shape of the rate-MTF curve (Fig 3B). Fixing the input vector strength (VS) at the maximum value (Fig 3B, gray curve) had only limited effects. Additional fixation of the input rate to the maximum value (Fig 3B, thin curve) led to a spike rate increase without much altering the curve shape. In this case, the output spike rate slightly increased for *f*_*m*_ > 1000 Hz, which resulted from the combination of the fixed high degree of phase-locking and the entry of the second modulation cycle into the 0.8-ms coincidence window. The phase-locking output of the model LSO was not affected by the *f*_*m*_-dependence on input rates or phase-locking (Fig 3C). In sum, the monaural AM-tuning of LSO was relatively insensitive to input parameters, which lends support to the suggestion that the observed low-pass properties of LSO neurons should originate from synaptic and membrane factors [36]. Possible effects of spontaneous inhibition will be examined separately in a later subsection.

#### Effects of coincidence threshold

**Fig 4:**
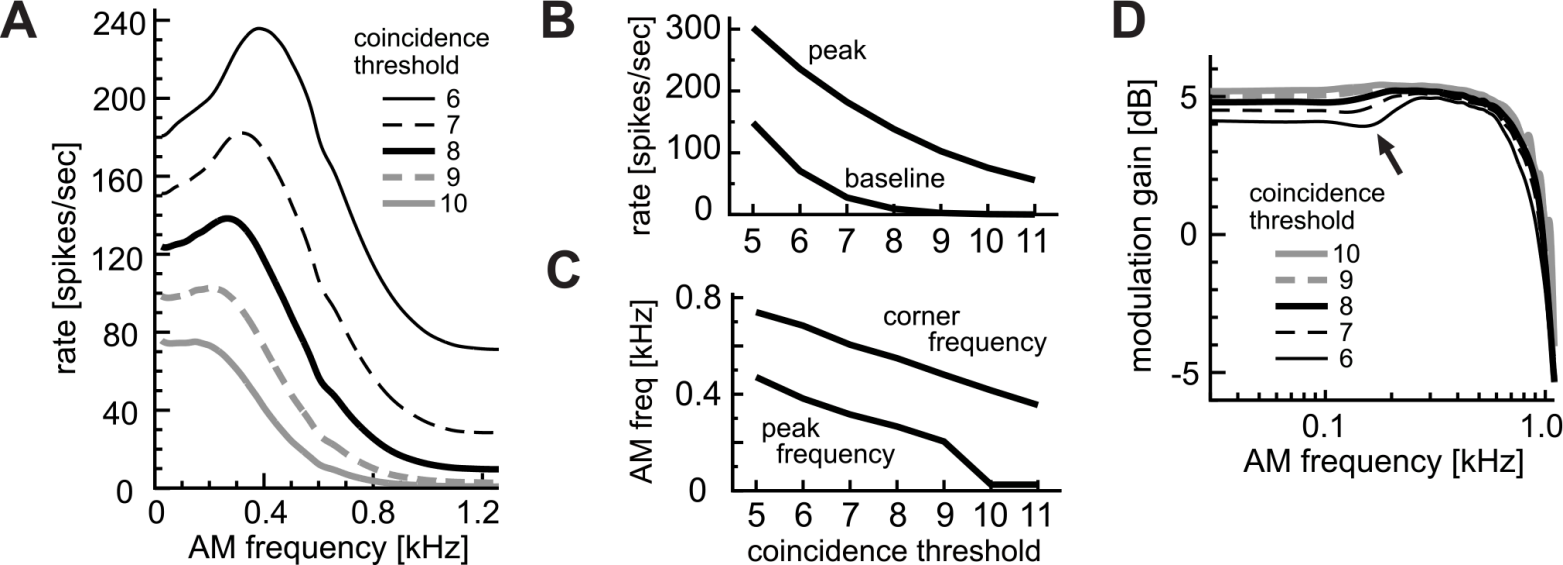
Effects of coincidence threshold θ. **A:** AM-tuning curves (rate-MTFs). **B:** Peak and baseline spike rates. **C:** Peak and corner frequencies of the rate-MTFs. **D:** Modulation gains (synch-MTFs). Line types in D correspond to those in A.

Fig 4 summarizes the effects of the coincidence threshold θ on monaural AM-tuning. The choice of coincidence threshold θ greatly affected the response rates of the modeled LSO neuron to AM sounds (Fig 4A). Decreased thresholds led to higher baseline rates as well as to higher peak rates (Fig 4A and 4B). With lower thresholds, peak and half-peak frequencies shifted to higher modulation frequencies (Fig 4A and 4C). For a high threshold (θ = 10 in Fig 4A), the AM-tuning curve was monotonic, with a peak frequency being close to zero Hz. Thus rate-MTF curves of LSO neurons with low thresholds tended to be shifted both to higher spike rates and to higher modulation frequencies. These observations suggest that LSO neurons with low rate-MTF (Fig 1A, orange lines) may have high coincidence thresholds, whereas those with high average spike rates over the entire frequency range (Fig 1A, green lines) may have low thresholds.

The modulation gain was higher for high coincidence thresholds (Fig 4D), consistent with the general assumption that a high threshold leads to better phase-locking with lower spike rates. For lower coincidence thresholds, we observed a small 'dip' in the synch-MTF (arrow in Fig 4D) associated to higher output rates, which was also seen experimentally in some LSO neurons [36].

#### Effects of coincidence window

**Fig 5:**
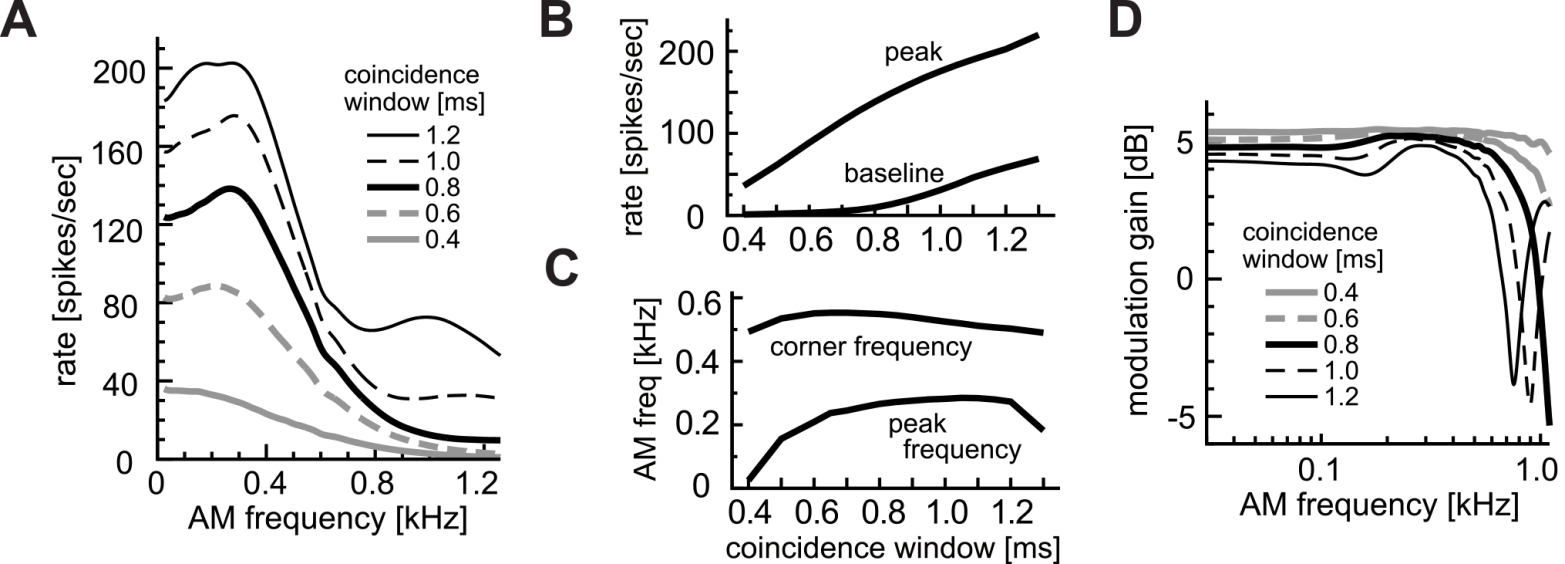
Effects of coincidence window W. **A:** AM-tuning curves (rate-MTFs). **B:** Peak and baseline spike rates. **C:** Peak and corner frequencies of the rate-MTF curves. **D:** Modulation gains (synch-MTFs). Line types in D correspond to those in A.

We next examined possible effects of the coincidence window on monaural AM coding (Fig 5). Changing the width W of the coincidence window led to variations in the shape of the rate-MTF curve (Fig 5A). Similar to the coincidence threshold, W affected the peak and baseline rates of AM-tuning curves (Fig 5A and 5B). In contrast, the peak and half-peak frequencies did not greatly change if W was in the range between 0.6 and 1.1 ms (Fig 5A and 5C). For a wide coincidence window (W = 1.2 in Fig 5A), a second peak appeared around 1000 Hz, because more than one modulation period lay in the coincidence window. In previous *in vivo* experiments, a few LSO units showed second peaks (Fig 1A, green lines).

The width W of the coincidence window affected both the amplitude and the cut-off frequency of the modulation gain (Fig 5D). A longer coincidence window led to a lower modulation gain and a lower cut-off frequency. The rebound of the modulation gain around 1000 Hz for large values of W arose because the simplified coincidence counting model did not incorporate low-pass properties of the membrane and synapses that limit high-frequency phase-locking.

In sum, variations in W could be related to the observed variations of the AM-tuning and modulation gain of LSO neurons. Low-rate units (Fig 1A, orange), for example, may have short coincidence windows, while high rate units (Fig 1A, green) may be associated with longer windows. Simulation results with coincidence windows of > 1.0 ms, however, were mostly inconsistent with empirical data, since second peaks in the rate-MTF curve were only infrequently observed and the modulation gain did not normally re-increase below 1000 Hz [36].

#### Combined effects of coincidence threshold and window

**Fig 6:**
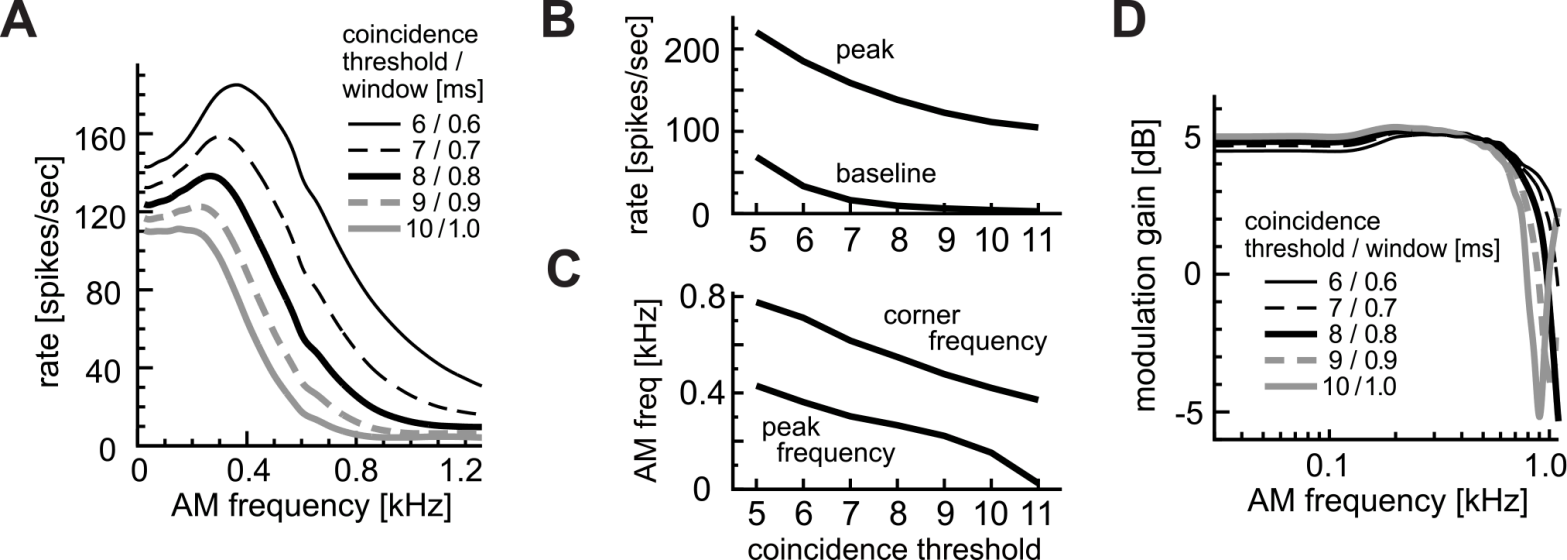
Combined effects of coincidence threshold θ and coincidence window W with a fixed ratio of θ/W. **A:** AM-tuning curves (rate-MTFs). **B:** Peak and baseline spike rates. **C:** Peak and corner frequencies of the rate-MTF curves. **D:** Modulation gains (synch-MTFs). Note that also for panels B and C, both the coincidence threshold and window were changed together as in panels A and D.

It was previously discussed that the height of the coincidence threshold θ and the width W of the coincidence window were, to a certain extent, interchangeable [43]. Yet in our simulations, these parameters showed different effects on the rate-MTF curves (Figs 4 and 5). To test this hypothesis more directly, we covaried θ and W while fixing their ratio constant (i.e., a higher threshold for a wider window). Simulated effects of these covaried parameters on the AM-tuning (Fig 6A) were qualitatively similar to the effects of the coincidence threshold (Fig 4A), rather than to the coincidence window (Fig 5A). Namely, both peak heights (Fig 6A and 6B) and frequencies (Fig 6A and 6C) changed according to the threshold. However, this parameter dependence was slightly milder, indicating that changes in W counteract the effects of θ. The synch-MTFs (Fig 6D) were almost insensitive to the combined variation of these parameters, with minor changes in the location of upswing due to the coincidence window. These results suggest that, when the ratio θ/W is fixed, the coincidence threshold would govern the overall tuning, while the width of the coincidence window may additionally modify the shape of the AM-tuning curves of LSO.

**Fig 7:**
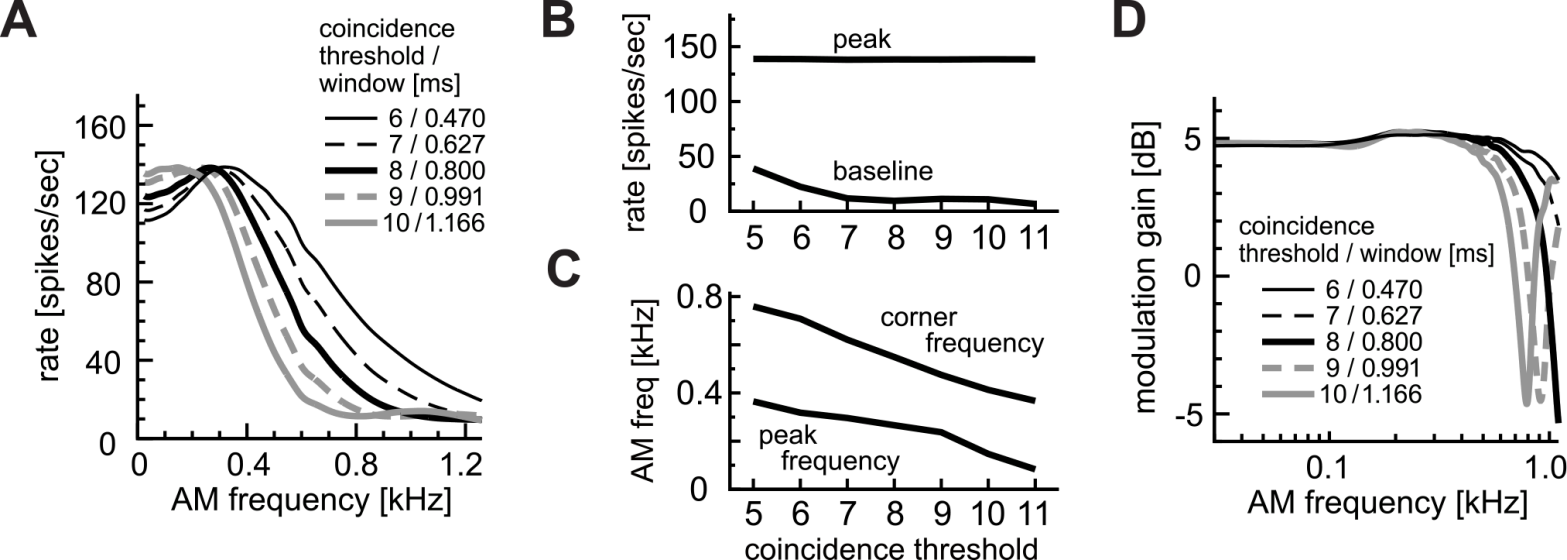
Combined effects of coincidence threshold θ and coincidence window W with fixed maximum spike rates. **A:** AM-tuning curves (rate-MTFs). **B:** Peak and baseline spike rates. **C:** Peak and corner frequencies of the rate-MTF curves. **D:** Modulation gains (synch-MTFs). Note that also for panels B and C, both the coincidence threshold and window were together changed as in panels A and D.

To further clarify the distinct effects of θ and W, we adjusted the size of W so that the peak spike rates were comparable to the default parameter settings (138–139 spikes/sec: Fig 7A). In this case, the peak and baseline rates were insensitive to parameter changes except for very low thresholds (Fig 7A and 7B). However, the peak and corner frequencies were greater for a lower threshold with a narrower coincidence window (Fig 7A and 7C). This is because a smaller θ results in a higher half-peak frequency (as seen in Fig 4), while the width W does not greatly affect these frequencies (as seen in Fig 5). Similar to Fig 6D, tails of the synch-MTF curves showed upswings because of large coincidence windows, whereas the low frequency part of the modulation gain did not show strong dependence on the parameter changes (Fig 7D). From these observations, we conclude that, at least in our model of LSO, the coincidence threshold θ and the coincidence window W are not simply interchangeable. The peak and half-peak frequencies are primarily determined by the coincidence threshold, not by the coincidence window. The threshold is a major determinant for the frequency limit of the rate-MTF curve.

#### Effects of refractory period

**Fig 8:**
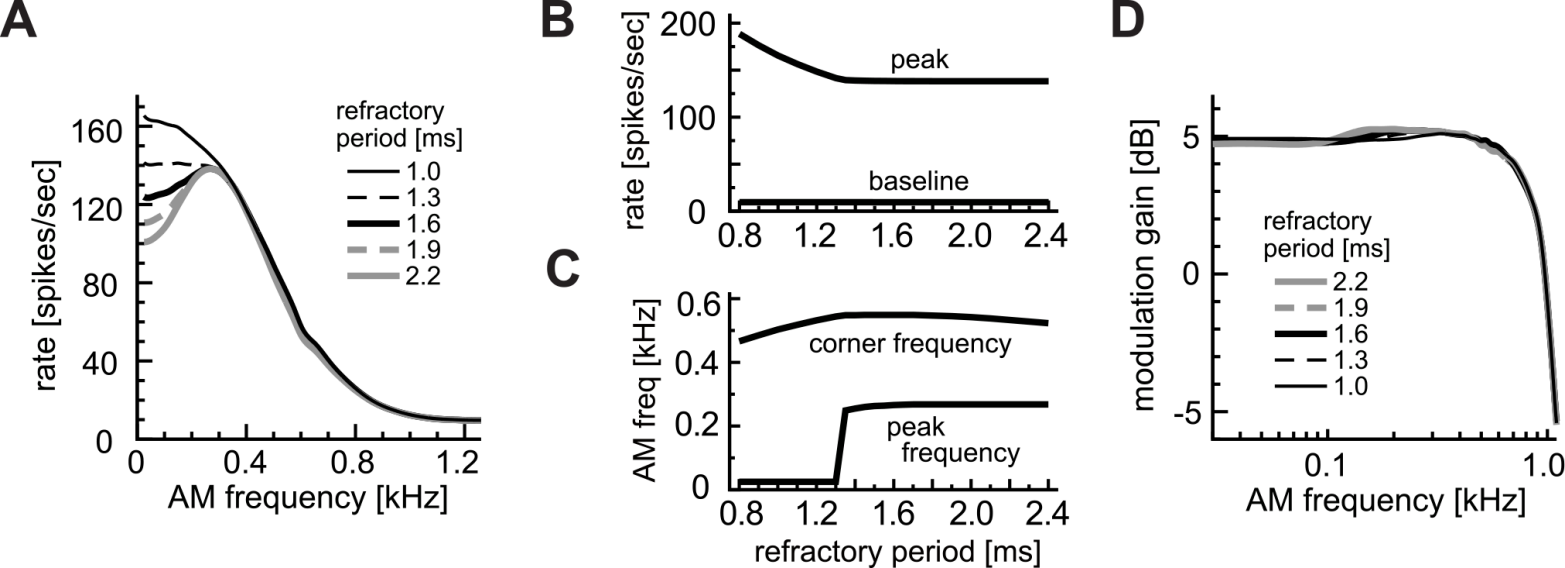
Effects of refractory period T. **A:** AM-tuning curves (rate-MTFs). **B:** Peak and baseline spike rates. **C:** Peak and corner frequencies of the rate-MTFs. **D:** Modulation gains (synch-MTFs). A jump in the peak frequency in C reflects transitions of AM-tuning between low-pass and band-pass. Line types in D correspond to those in A.

In contrast to the coincidence threshold θ and window W, which affected the shape of the AM-tuning curve over the entire range, simulated effects of the refractory period T were limited to low modulation frequencies (Fig 8A). Variations of T affected the shape of the AM-tuning curve only below 300 Hz. For these low modulation frequencies, which are considerably lower than the half of the reciprocal of the coincidence window, two or more input coincidences could occur within the same modulation period, but because of the refractory period, not all coincidences led to output spikes. Hence a longer refractory period resulted in lower rates at low modulation frequencies without affecting the baseline (Fig 8A and 8B) and the corner frequency (Fig 8A and 8C). These observations suggest that a salient peak in the rate-MTF curve may be related to a refractory period > ~1.4 ms, and that monotonically decreasing rate-MTF (Fig 1A, red lines) may originate from a shorter refractory periods. As expected from the rate-MTF, the synch-MTF for *f*_*m*_ > 300 Hz was not affected by the refractory period (Fig 8D).

A high coincidence threshold or a short coincidence window led to fewer coincidence counts (e.g., θ = 10 in Fig 4A and W = 0.4 ms in Fig 5A). In such a case, effects of the refractory period were generally weak, resulting in a more monotonic rate-MTF curve. Considering that the subthreshold membrane properties of LSO neurons are generally low-pass except for low CF neurons [19], the band-pass property of the rate-MTF should originate from the suprathreshold activity of the neuron. Our modeling results suggest that a relatively long refractory period combined with fairly high output rates (typically > 100–150 spikes/sec) may be relevant to the band-pass rate-MTFs, which were frequently observed in experiments (Fig 1A, blue curves).

#### Effects of spontaneous inhibition

**Fig 9:**
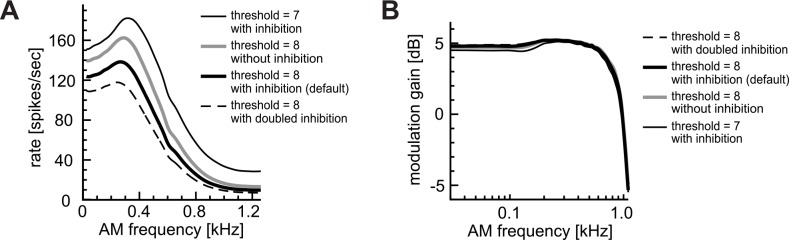
Effects of spontaneous inhibitory inputs. **A:** AM-tuning curves (rate-MTFs). **B:** Modulation gains (synch-MTFs). Curves for θ = 8 (default threshold: thick lines) and 7 (reduced threshold: thin line) are shown for comparison. Spontaneous rates *λ*_inh_ of inhibitory inputs were: 0 (no inhibition), 30 (default) and 60 (doubled inhibition).

MNTB neurons that send inhibitory projections to LSO are spontaneously active [23,50]. Effects of spontaneous inhibition on the AM-tuning curve, however, were found to be limited (Fig 9A). Removal of inhibition only resulted in a slight increase in the rate of response to monaural stimulation. Simple calculations may explain these limited effects of inhibition. Assuming that an LSO neuron receives 8 inhibitory inputs with a spike rate of 30 spikes/sec and that each inhibitory input elevates the threshold by 2 with an effective time window of 1.6 ms, the time-averaged threshold increase can be estimated as: 8×30×1.6×10^−3^×2 = 0.768 inputs. Therefore the average effect of spontaneous inhibition is expected to be smaller than the effect of changing the threshold by one.

Even when the spontaneous rates of inhibition were doubled, the overall shape of the rate-MTF did not significantly change (Fig 9A, broken line). Similarly, the modulation gain was not affected by the spontaneous inhibition (Fig 9B), whereas coding of binaural phase differences essentially relies on inhibition driven by contralateral stimulus sounds (Fig 2E). These results suggest that spontaneous inhibition should play only a marginal role (if any) in monaural coincidence detection, compared with the significant role of sound-evoked inhibition in binaural phase-coding of AM sounds.

### Binaural AM Coding in LSO

#### Coincidence parameters

In the preceding sections, we have examined how variations of coincidence detection properties affect monaural AM-tuning in the model LSO. Since the LSO is one of the earliest locations where inputs from the two ears converge, binaural AM coding of LSO in terms of coincidence detection should also be examined. In this and following subsections, we test how variations in the model parameters of coincidence detection may play a role in binaural phase-coding of AM sounds. We fixed the AM frequency at *f*_*m*_ = 300 Hz, where the model LSO strongly responded to monaural AM stimuli (Fig 2D). We here focus on the peak and trough spike rates as well as the half-peak width and the trough phase (see Fig 2E for their definitions), because the location and the width of the trough of a phase-response curve provide fundamental information on the temporal tuning of an LSO neuron that receives ipsilaterally driven excitatory inputs and contralaterally driven inhibitory inputs (e.g., [28,29]).

**Fig 10:**
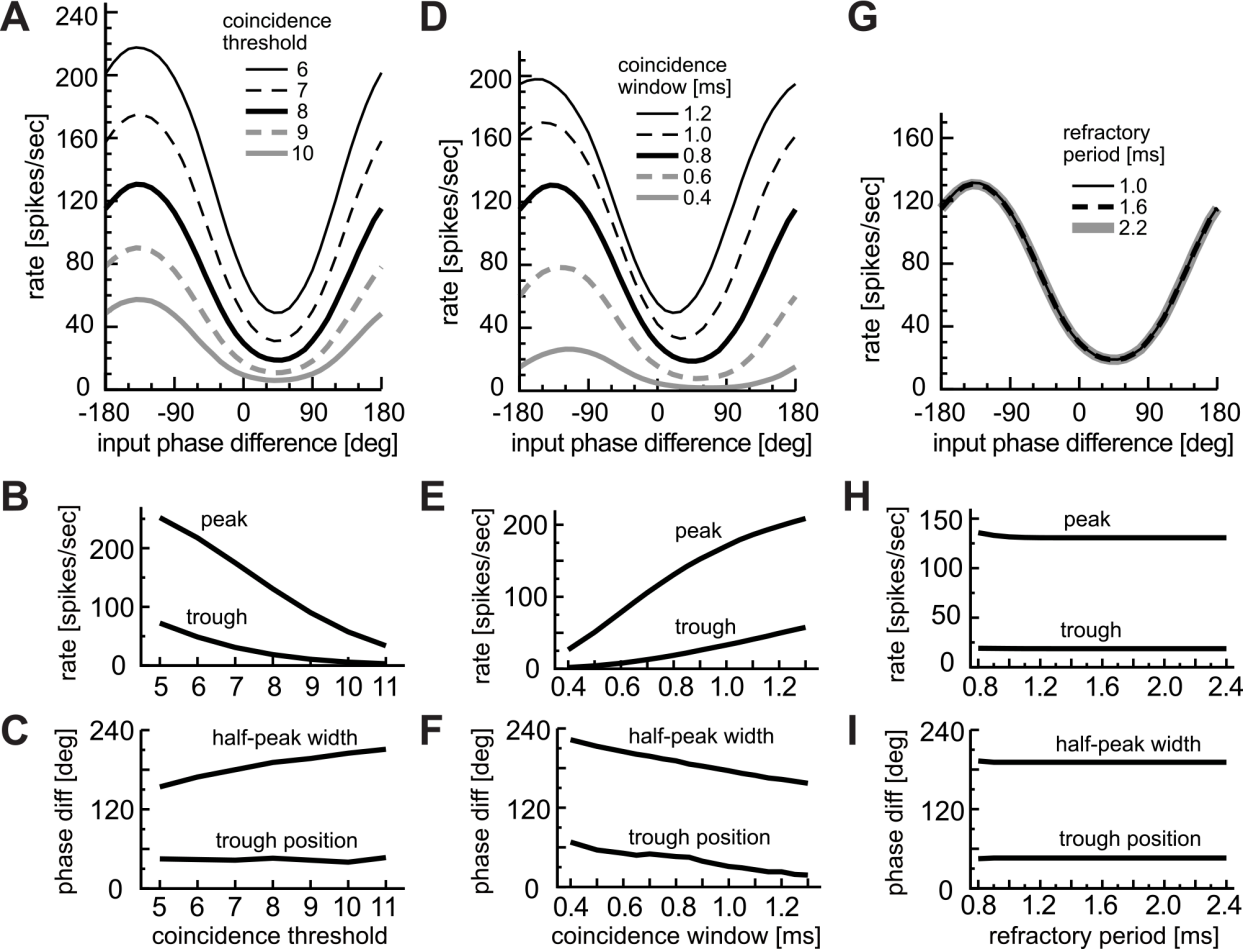
Effects of coincidence detection parameters on binaural phase coding. **A-C:** Effects of the coincidence threshold θ. **D-F:** Effects of the coincidence window W. **G-I:** Effects of the refractory period T. **A,D,G:** Phase-tuning curves. **B,E,H:** Peak and trough rates of the phase tuning curves. **C,F,I:** Half-peak width and trough phase of the phase tuning curves.

We first varied the coincidence threshold θ (Fig 10A–10C). As naturally expected, the overall spike rates were higher for low thresholds (Fig 10A and 10B), but the location of the trough phase did not change with the threshold (Fig 10A and 10C). The half-peak width tended to be narrower for deeper phase-tuning curves (Fig 10C).

Second, we examined the effects of the width W of the coincidence window (Fig 10D–10F). A large W resulted in both an increased spike rate (Fig 10E) and a more centered phase-response curve with a trough phase located near zero degrees (Fig 10D and 10F). Therefore, the value of W was a major determinant of the trough phase (Fig 10F). This suggests that different mechanisms may determine the binaural phase-tunings (at fixed frequency) and the monaural *f*_*m*_-tunings; i.e., the peak and corner frequencies of the monaural rate-MTF is affected primarily by the coincidence threshold θ (Figs 4–7), while the trough position of the binaural phase-tuning curve depends greatly on the coincidence window W.

Next, effects of the refractory period T were tested (Fig 10G–10I). Simulation results indicated that, at *f*_*m*_ = 300 Hz, the length of T does not affect the binaural phase-tuning curves. This suggests that possible variations of the refractory period may not be reflected to the variations of binaural phase response properties of real LSO neurons, except for very low (< 200Hz) modulation frequencies.

#### Inhibitory inputs driven by contralateral sounds

Our simulation results showed that spontaneous inhibitory inputs had only limited effects on monaural AM tuning (Fig 9). In binaural phase coding, however, sound-driven inhibition may play a more active role. To test this hypothesis, we varied the inhibitory model parameters; namely, the threshold increase δ and inhibition window Δ (see Materials and Methods for definitions).

**Fig 11:**
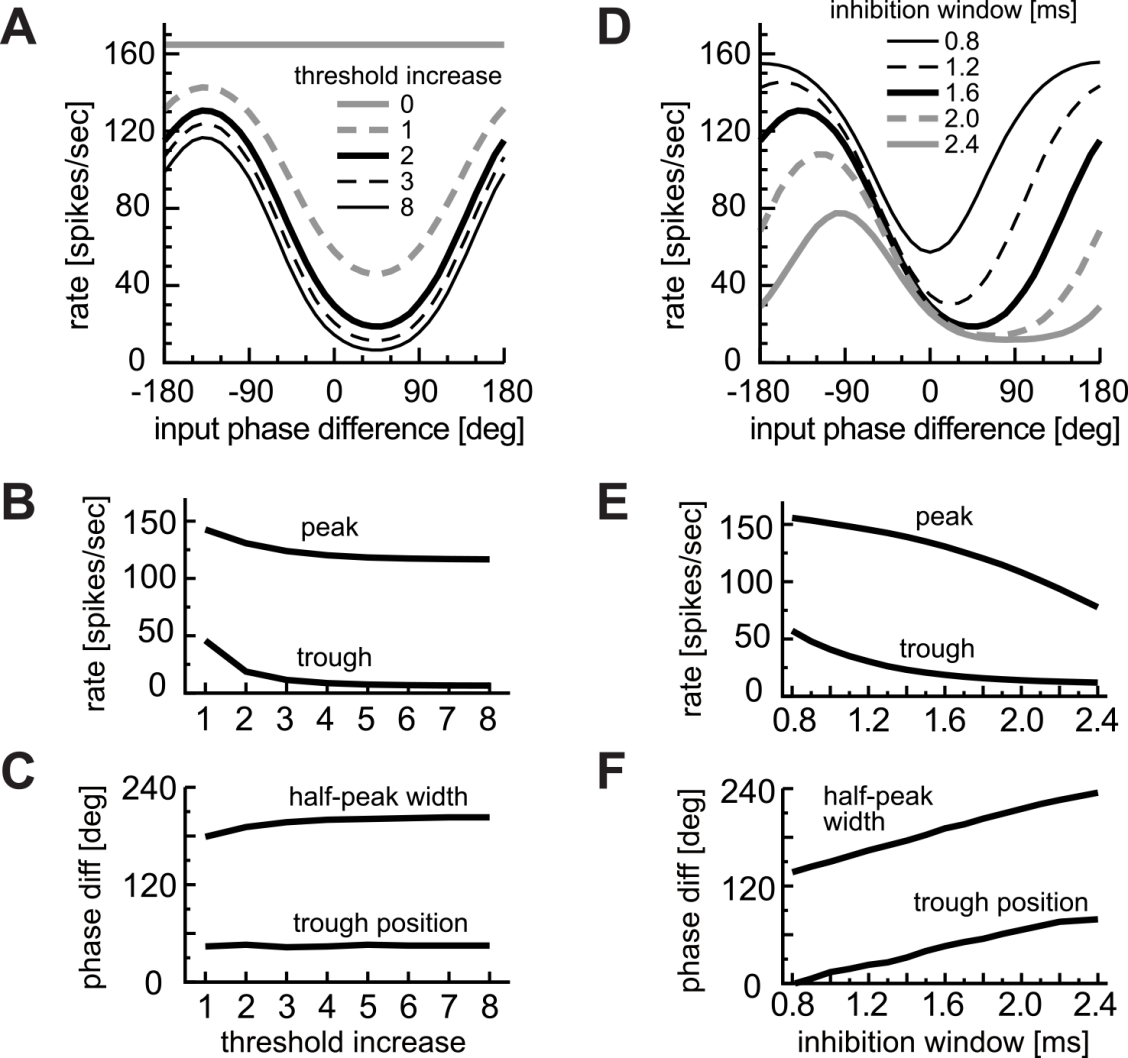
Effects of inhibition parameters on binaural phase coding. **A-C:** Effects of the threshold increase δ. **D-F:** Effects of the inhibition window width Δ. **A,D:** Phase-tuning curves. **B,E:** Peak and trough rates of the phase tuning curves. **C,F:** Half-peak width and trough phase of the phase tuning curves.

The effect of the threshold increase δ was most prominent when it was varied from zero (i.e., no inhibitory effects) to one or two, resulting in a transition from a flat to standard phase-response curve with a distinct peak and trough (Fig 11A). Further changes in threshold increase did not greatly affect the phase-coding (Fig 11A and 11B), indicating that effects of inhibition do not need to be extremely strong to create phase-sensitivity in LSO. Moreover, the threshold increase was generally ineffective on determining the half-peak width and the trough location (Fig 11C).

Variation in the width Δ of the inhibition window showed more dynamic effects on phase-response curves (Fig 11D). A narrow inhibition window led to a higher spike rate (Fig 11E) and to a trough phase closer to zero degrees (Fig 11F). Thus both the inhibition Δ (Fig 11D–11F) and the coincidence window W (Fig 10D–10F) had comparable effects on determining the trough phase and width of the binaural phase-tuning curve, but the directions of their effects were opposite. In sum, our simulation results suggest that the overall rates of the phase-response curve were determined primarily by the coincidence threshold θ, coincidence window W and the inhibition window Δ, whereas the trough phase was determined only by a combination of the latter two. Combined effects of these window parameters will be further examined in the next section.

#### Window sizes and trough positions

**Fig 12:**
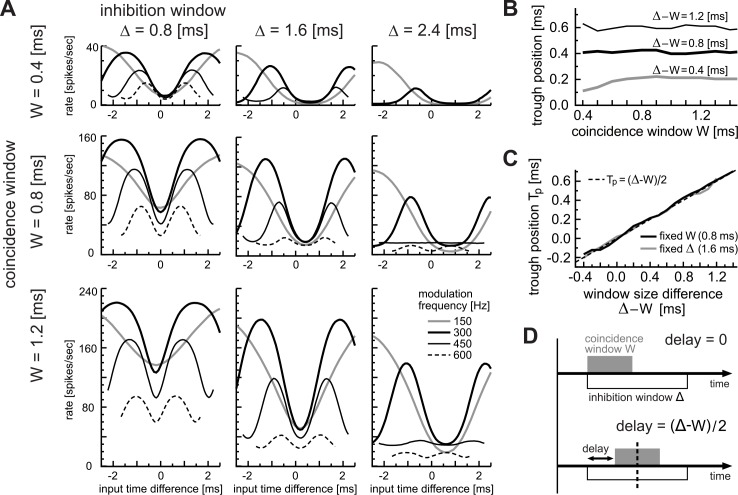
Effects of window parameters on binaural temporal coding. **A:** Combined effects of coincidence window W and inhibition window Δ. Phase-tuning curves for simulated AM inputs at 150, 300, 450 and 600 Hz are shown. Input phase differences (abscissa) are converted into milliseconds to facilitate comparison across frequencies. Different rows and columns correspond to different widths of the coincidence window W (0.4, 0.8, and 1.2 ms) and inhibition window Δ (0.8, 1.6, and 2.4 ms), respectively. **B:** Dependence of trough positions on covaried coincidence window W and inhibition window Δ. These window parameters were varied while their difference Δ-W was fixed at 0.4, 0.8 or 1.2 ms. **C:** Dependence of trough positions P_T_ on the window size difference Δ-W. Either of the window parameters was fixed at the default value (black: W = 0.8 ms; gray: Δ = 1.6 ms), while the other parameter was varied. The dotted diagonal line shows a slope of 0.5 (i.e., P_T_ = (Δ-W)/2). In **B** and **C**, the input modulation frequency was fixed at 300 Hz. **D:** Schematic drawing of how the inhibition window interacts with the coincidence window.

Fig 12A shows phase-tuning curves for different coincidence windows W, different inhibition windows Δ and at different modulation frequencies *f*_*m*_. A high modulation frequency generally led to lower spiking rates, which was shown in previous *in vivo* recordings (Fig 1B). Overall response rates were higher for larger coincidence windows and shorter inhibition window. The period of each phase-tuning curve corresponded to the reciprocal of the modulation frequency, and most importantly, the trough positions did not change with *f*_*m*_ (compare the model results of Fig 12A with experimental results of Fig 1B). Only exceptions were the phase-tuning curves for high modulation frequency combined with a wide inhibition window (Δ = 2.4 ms and *f*_*m*_ = 600 Hz: right column of Fig 12A), where the inhibition window covered more than a single modulation cycle. The trough positions varied with the window widths, as expected from previous results (Figs 10F and 11F). The phase-tuning curve was shifted towards more positive time differences by smaller W and larger Δ. When the two window widths took the same value (W = Δ = 0.8 ms: middle-left panel in Fig 12A), the trough was located at zero input time difference. In sum, all phase-tuning curves for different modulation frequencies aligned at the trough, whose position was dependent on the window widths W and Δ.

Our observations suggest that the trough position may be described as a function of these window widths. Therefore we plotted the trough positions with varied W while the difference Δ-W was fixed (Fig 12B). Under this condition, simulated trough positions were generally insensitive to W, except for very short coincidence windows (W<0.5 ms). This implies that neither of the window width alone, but their relative size should determine the trough position. To test this hypothesis, we plotted the location of the trough with the window size difference Δ-W (Fig 12C). Simulated trough positions (solid lines) clearly matched the straight line of a slope 0.5. Thus the trough position of a phase-tuning curve can be predicted by the half of the size difference of the coincidence and inhibition windows, namely (Δ-W)/2. A simple explanation is given in Fig 12D. When the excitatory and inhibitory inputs arrive with a zero delay (Fig 12D, top), the total effects of the coincidence and inhibition windows are asymmetric in time, because the width of the inhibition window was generally longer. A temporal symmetry of these window functions was achieved when the delay equals half of the width difference (Fig 12D, bottom). In the real nervous system, however, effects of these window functions may not be entirely uniform in time; e.g., synaptic inputs usually show a rapid increase and a slower decrease [51]. Nevertheless, these simulation results suggest that relative time scales of excitatory and inhibitory inputs can be a factor to determine the trough position of a phase-tuning curve.

#### Effects of interaural level differences

**Fig 13:**
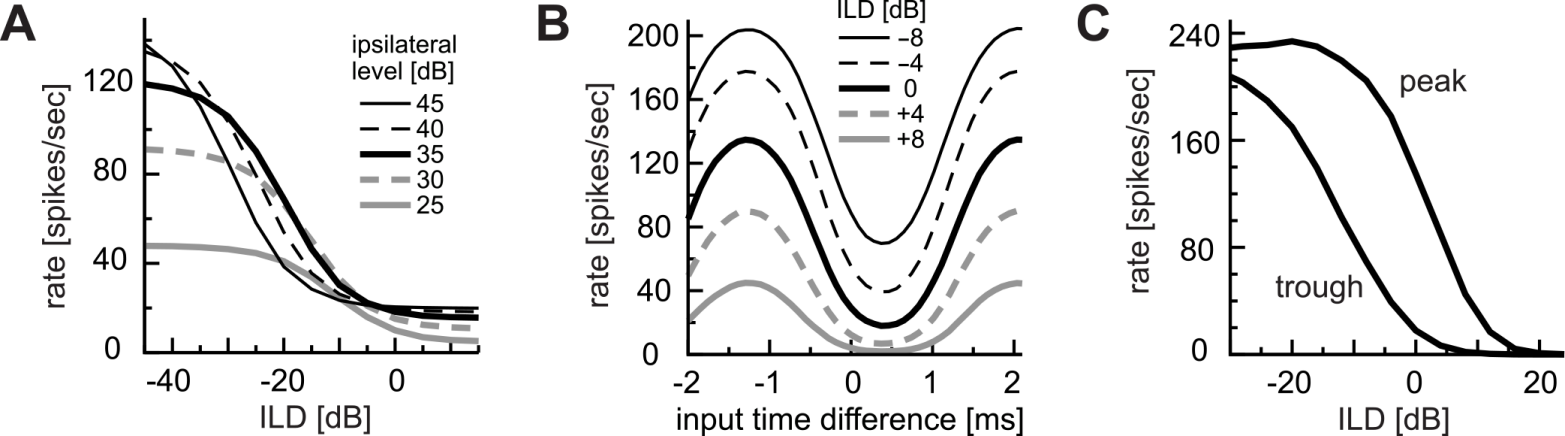
Effects of level difference on binaural temporal coding. **A.** Simulated output spike rate of the model LSO neuron for non-modulating inputs (*f*_*m*_ = 0 Hz). The ipsilateral sound pressure driving excitatory inputs was fixed at five different levels (25–45 dB) and the contralateral level was varied. **B.** Phase-tuning curves for different ILDs. **C.** ILD-dependence of the peak and trough spike rates of the phase-tuning curves. In **B** and **C**, the input modulation frequency and the average binaural level (defined as the arithmetic mean of the bilateral sound input levels) were fixed at 300 Hz and 20 dB, respectively, which corresponded to an input rate of 150 spikes/sec at ILD = 0.

In the preceding sections, we investigated monaural and binaural AM coding in the LSO with fixed sound stimulus levels. Since LSO neurons are sensitive to binaural level differences, we here examine how ILDs affect the output of our coincidence counting model. We varied ILDs of non-modulating inputs (Fig 13A). Simulated spike rates at different sound pressure levels showed classical ILD-tuning with higher spike rates for more negative ILDs (see [5] for related *in vivo* data using tonal inputs).

We next varied ILDs and calculated model outputs to input phase differences (Fig 13B). More negative ILDs (i.e., higher level for excitatory inputs than for inhibitory inputs) led to higher spike rates, but the trough position and the overall phase-tuning property were not affected. Furthermore, the model LSO showed phase-tuning properties for a wide range of ILDs (Fig 13C). These results closely match previous *in vivo* recording data from the cat LSO (see Fig 8 of [27]), suggesting that coincidence detection is a fundamental operation in both ILD-coding and in phase-coding of AM sounds.

### AM Coding of Pure Integrator

Our simulation results suggest that temporal processing of excitatory and inhibitory inputs is essential for both monaural and binaural AM-tunings. To further confirm the importance of coincidence detection in LSO, we performed additional simulations using a pure integrator model fed with the same set of inputs as used for the coincidence counting model. The perfect integrator model does not have a coincidence window or an inhibition window, since it simply sums excitatory inputs and subtracts inhibitory inputs regardless of their timings (see Materials and Methods).

**Fig 14:**
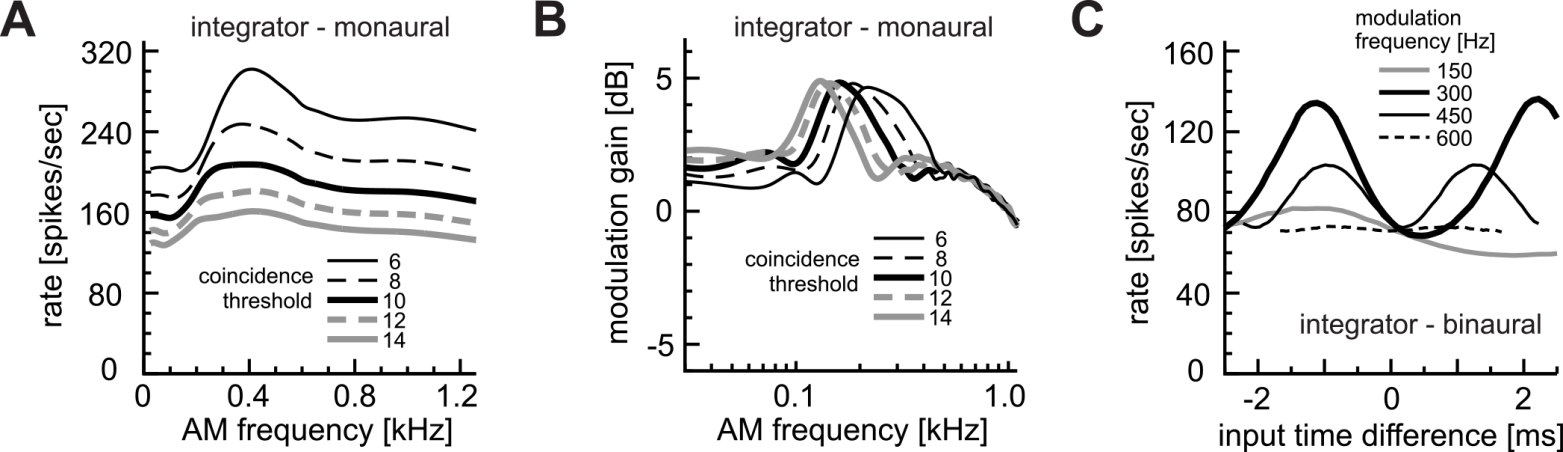
Response of pure integrator model. **A:** Simulated monaural AM-tuning curves (rate-MTFs). **B:** Modulation gains (synch-MTFs). **C:** Binaural phase-tuning curves at different frequencies. The same input parameter set as for Fig 2D and 2E was used. The threshold was fixed to 8 inputs in C.

Simulated rate-MTF curves of the pure integrator showed a broad tuning (Fig 14A), which resembles the all-pass property of input fibers (Fig 2A). The response rates were slightly reduced at low frequencies (< 300 Hz) where the refractory period plays a role. Simulated synch-MTFs of the pure integrator showed a high gain (> 3 dB) for only a narrow range of modulation frequencies (Fig 14B). For other frequencies, gains were generally lower than the coincidence counting model (Fig 4D) and physiological data [36]. Thus the responses of the pure integrator were largely inconsistent with empirical LSO responses to monaural AM sounds (Fig 1A and [36]).

For binaural inputs, the pure integrator model also showed sensitivity to time differences of excitatory and inhibitory inputs (Fig 14C). Although the pure integrator lacks explicit time windows, spike generation (and subsequent refractory period) imposes an effective time limit of synaptic integration, since the model neuron resets its state after each spike. Namely, for an output rate of 200 spike/sec, for example, integration of inputs must occur on the average time scale of 5 ms. In other words, even in the pure integrator, thresholds are more likely to be reached when excitatory inputs arrive earlier than inhibitory inputs, whereas early inhibition simply cancels late excitation which leads to fewer output spikes. A similar excitation-inhibition interaction was reported in a modeling study that used a slow integrator [58]. Nevertheless, simulated trough positions varied with modulation frequency (Fig 14C), indicating that simulated responses of the pure integrator did not match empirical binaural LSO responses (Fig 1B).

## Discussion

### (Anti-)Coincidence Detection in LSO

Amplitude modulation is a common feature of natural stimuli, and hence processing of AM signals is important in auditory [31] and other sensory systems (e.g., [59,60]). In the present study, we used a simple coincidence counting model of LSO neurons and investigated how monaural and binaural AM coding is affected by several biophysical factors. The model has a small number of abstract parameters for coincidence detection, which may be controlled by lower-level physiological mechanisms (i.e., detailed properties of the membrane and synapses). By changing the coincidence parameters of the model, we reproduced most of rate- and synch-MTF curve variations to monaural AM sounds observed *in vivo* [36]. Furthermore, the same modeling conditions were largely sufficient for explaining the binaural temporal coding in the LSO. Unlike the coincidence counting model, the pure integrator model failed to reproduce monaural and binaural AM responses of LSO. These results suggest that coincidence detection is a fundamental operation in the LSO for both monaural and binaural auditory information processing. Previous modeling results that showed possible roles of spike-timing dependent plasticity in LSO also support this suggestion [61].

Octopus cells in the posteroventral cochlear nucleus and principal cells of the MSO are also known to act as auditory coincidence detectors [62]. The integration window of the octopus cell is ~1 ms [63], whereas the coincidence window of the MSO neuron is typically a few hundred microseconds or even shorter [43,64]. In our series of simulations, results were mostly consistent with empirical observations when we set the width of coincidence window at 1.0 ms or less. This value is comparable to the time scale of coincidence detection in octopus cells and even shorter than the integration time windows of cortical coincidence detectors, which are on the order of a few milliseconds [65].

Monaural coincidence detection has been studied frequently in relation to the improvement of temporal coding performance at the cochlear nucleus [55,66,67], which is sending excitatory afferents to the LSO. A prior modeling study showed non-monotonic AM-tuning curves and improved synchrony in auditory midbrain neurons where excitatory and inhibitory inputs converge [54]. Our LSO model also exhibited improved phase-locking for a wide range of parameters.

The parameters of excitatory inputs (i.e., rate and degree of phase-locking), which depended on the modulation-frequency, had only limited effects on monaural AM coding (Fig 3). Spontaneous inhibition also showed minor effects on the shape of monaural rate-MTFs (Fig 9). In contrast, the coincidence parameters, such as the threshold (Fig 4) and the window width (Fig 5), both affected the height and the shape of the rate-MTF curve but in different manners. Peak and corner frequencies were more strongly affected by the coincidence threshold than by the coincidence window. The refractory period (Fig 8) showed its effect only on the low-frequency portion of the rate-MTF. These results support the hypothesis [36] that synaptic and postsynaptic coincidence factors should play an essential role in determining monaural AM-tuning properties, while presynaptic input properties have only minor impacts. Increases in coincidence threshold or decreases in width of the coincidence window resulted in higher gain with lower spike rates (Figs 4 and 5). Existence of such relations *in vivo* needs to be tested in future experiments.

In contrast to monaural AM-tuning curves, binaural phase-response curves showed distinct dependence on the model parameters. Both the coincidence threshold and window affected the LSO output rates to simulated binaural inputs, but the trough position of a phase-response curve was not sensitive to the threshold but to the window width (Fig 10). Moreover, the inhibition window was found to be another important factor that determines the location of the trough (Fig 11). Our simulation results suggest that the size difference of these windows was a major factor for determining the trough location of a phase-tuning curve (Fig 12). Therefore, in monaural AM coding, the coincidence threshold determines the limiting modulation frequency (i.e., the half-peak frequency), whereas in binaural AM coding, the temporal factors (coincidence and inhibition windows) have more direct impact on determining the phase-tuning properties. Our observations lend support to the suggestion that the relative latency of the excitatory and inhibitory inputs directly affects the output of LSO neurons [9,27]. Furthermore, our minimalistic coincidence counting model reproduced the classical ILD-tuning curve (Fig 13A) and the empirical ILD-dependence of phase-tuning curves (Fig 13B). These results indicate that the LSO neuron acts as an 'anticoincidence detector' where the comparison of excitatory and inhibitory synaptic inputs occurs in a (sub)millisecond time scale to compute 'instantaneous ILDs'. Similar anticoincidence detection was reported in the sensory system of electric fish [68,69].

### Sources of Variations

In agreement with our results, previous experimental studies reported comparable variations in coincidence detection parameters across neurons. The refractory period of cat LSO neurons *in vivo*, for example, ranged from 1.1–2.8 ms [52]. Similarly, mouse LSO neurons recorded *in vitro* showed variations in the refractory period [70]. As previously suggested, membrane afterhyperpolarization and sodium channel inactivation would play major roles in determining the refractory period [18]. The number of subthreshold inputs of the gerbil LSO neurons measured *in vitro* also showed considerable variations (9.6 ± 2.8 excitatory inputs)[51]. The width of the coincidence window is determined by multiple factors [43], such as the synaptic and membrane time constants [62], inhibitory inputs (e.g., [71]) and dendritic processes [72]. Excitatory and inhibitory synaptic inputs, for example, showed neuron-to-neuron variability in both time scale and amplitude [51], which should be related to the model parameters W and θ. Empirically measured membrane time constants also showed large variations (1.1 ± 0.4 ms in gerbils [51]; 1.9–4.1 ms in mice: [70]; and 8.5 ± 4.5 ms in rats [73]). A previous theoretical study demonstrated that, under the assumption of linear summation of synaptic conductances and linear membrane responses, the width of coincidence window is linear to the membrane time constant [74].

The KLVA conductance, a major determinant of the resting membrane properties (e.g., [75]), shows graded distributions along the tonotopic axis in the LSO with neurons in the high-frequency medial limbs of the LSO showing the lowest density [76]. Increases in KLVA conductance reduce the resting input impedance and shorten the membrane time constant, which would result in a higher coincidence threshold and a shorter coincidence window [62]. Interplay between KLVA and sodium inactivation may further narrow the coincidence window [77]. In addition, KLVA conductance plays a major role in sensing the rising slope of synchronized synaptic inputs (VCN [78]; MSO [79]). Future dynamic clamp experiments *in vitro*, for example, might reveal more detailed relations between the higher-level coincidence parameters and the underlying membrane and synaptic factors. Previous studies showed that having non-homogeneous neuronal population is often beneficial for coding sensory information [59,80,81]. The mammalian LSO might also benefit from the observed heterogeneity of coincidence detection properties that may enhance the information capacity of the neuronal population.

### Relevant Modeling Approaches, Applications and Limitations

Our minimalistic coincidence counting model has only a small number of parameters (Table 1), which made our analyses simple. As has been repeatedly discussed [82–84], there is always a trade-off in modeling studies between simplicity and realism. Simple models are useful for describing the operational principles of a complex system and suitable for large-scale simulations that are computationally demanding. It should also be noted that complex (~realistic) models do not always produce physiologically more accurate results compared to simpler models [85].

In our phase-tuning curves (Figs 10–12), model LSO spike rates were plotted against phase (or time) difference of simulated synaptic inputs. In the actual auditory system, external acoustic delays (i.e., interaural time differences) are combined with internal delays (such as cochlear, axonal, and other delays) to determine the relative synaptic input timings of the target binaural neuron [1,40]. Previous cat LSO studies hypothesized that, if the external delays are perfectly compensated by the internal delay, the trough position should be equal to zero [27,28]. Our simulation results showed that the relative sizes of the coincidence and inhibition windows may play an additional role in determining the trough position (Fig 12C). A similar assumption was claimed in a study of MSO that a binaural mismatch in excitatory synaptic time constants could be a further source of delays [86], although this hypothetical mechanism was later denied by their following study that reported symmetric synaptic inputs in the MSO [87]. Nevertheless, since inhibitory synaptic inputs are significantly slower than excitatory inputs in the LSO [51], mismatches in temporal properties of ipsi- and contralateral synaptic inputs may still be an essential factor to determine the binaural tuning of LSO. Shifts of peak frequencies induced by timed inhibition were also suggested by a number of modeling studies of binaural neurons in the MSO (e.g., [88,89]).

A prior modeling study suggested that a large amount of KLVA conductance was necessary to reproduce empirical AM-frequency dependence of LSO spike rates [39]. The majority of experimental data on AM coding in LSO neurons, however, comes from high-frequency sensitive neurons with low expression of KLVA. Our results indicate that various other factors related to the coincidence parameters should also play an essential role in determining the rate-MTF. Temporal coding properties of the neuron may be affected by, for example, spatial distributions of sodium channels [90] and interactions between KLVA and hyperpolarization-activated cation currents [91]. Furthermore, summation of synaptic inputs along the bipolar dendrites of LSO [92] should also affect coincidence detection [72,93].

The coincidence counting model we used was originally introduced for investigating the binaural coding in the MSO [43]. This model can be regarded as an extension of the binaural 'shot-noise' model, in which excitatory inputs are added and inhibitory inputs are subtracted to calculate a model spike output [14,16]. Such shot-noise models share basic response characteristics to a leaky integrate-and-fire model with a short membrane time constant (e.g., [94,95]). Interchangeability of the coincidence threshold and window was suggested in MSO [43]. Our LSO simulation results, however, indicated that they have considerably different effects on AM tunings (Figs 6, 7, and 10). This would be related to the difference in operational time scales of MSO and LSO and may imply their functional differences as asserted by Remme et al. [19].

Although our results were consistent with previous empirical data [27,28,36], most of the limitations that Franken et al. [43] discussed also apply to our approach. For example, the model often shows better phase-locking at high modulation frequencies (> 1000 Hz) than empirical data, because it lacks additional factors that may limit the temporal precision of neuronal response (such as low-pass membrane, dynamical spike initiation, subthreshold potential fluctuation, etc.). This restricts the applicability of the model to the frequency range below 1000 Hz. Furthermore, we simply increased the coincidence threshold to mimic the effects of inhibitory inputs. The actual effect of inhibition, however, may be subtractive or divisive (e.g., [96]) depending on whether it is of hyperpolarizing and/or shunting nature (see also [1] for discussion on subtractive inhibition in LSO). Spontaneous inhibition to the LSO from the ipsilateral MNTB was suggested to result in reduced monaural LSO firing rates at high *f*_*m*_ rates [39], but only for vastly unrealistic numbers of inhibitory inputs to LSO. Moreover, responses of LSO neurons may be modulated by ipsilaterally driven inhibitory inputs from the lateral nucleus of the trapezoid body (LNTB) and also by inhibitory inputs from the ventral nucleus of the trapezoid body (VNTB) that receives bilateral afferents from cochlear nuclei and efferents from higher auditory stages [97,98]. Time-delayed ipsilateral inhibition from LNTB is suggested to affect the discharge patterns of LSO neurons [99]. How inhibitory inputs affect the coincidence detection in the real LSO could be investigated by examining subthreshold membrane responses with whole-cell, *in vivo* methods, which were recently used for MSO neurons [64].

Our LSO model consisted of two parts: (1) generation of time-locked excitatory and inhibitory inputs and (2) coincidence detection of the LSO neuron. As was done in a previous modeling study [19], sound-driven synaptic inputs can be replaced with a more detailed model that closely represents activities of the auditory periphery [100]. Testing with more complex acoustic stimuli (such as vocalization or speech [19,31]) or with transient stimuli (such as clicks [27,30]) would further reveal the applicability and limitations of the coincidence counting model. Recent neurophysiological recordings *in vivo* showed that coincidence detection in MSO is affected by its input history [64]. Similar dynamical threshold adaptation may also exist in the LSO. Further studies are necessary to reveal how monaural and binaural AM-tuning properties of LSO neurons eventually result in the sound localization behavior of the animal.
